# Coixol and Sinigrin from *Coix lacryma-jobi* L. and *Raphanus sativus* L. Promote Fat Browning in 3T3-L1 Adipocytes

**DOI:** 10.3390/ph18121843

**Published:** 2025-12-02

**Authors:** Seung Min Choi, Sung Ho Lim, Ho Seon Lee, Gayoung Choi, Myeong Ji Kim, Hyunwoo Kim, Chang-Ik Choi

**Affiliations:** BK21 FOUR Team and Integrated Research Institute for Drug Development, College of Pharmacy, Dongguk University-Seoul, Goyang 10326, Republic of Korea; tlsehdhs@dgu.ac.kr (S.M.C.); sho617@dgu.ac.kr (S.H.L.);

**Keywords:** thermogenesis, natural compounds, lipid metabolism, beige adipocytes

## Abstract

**Background/Objectives**: Obesity, a metabolic disorder resulting from an energy imbalance, often leads to excess fat and related diseases. Browning of white adipose tissue, which increases energy expenditure, is a promising anti-obesity strategy. Herbal medicines are considered safer than conventional drugs, but their fat browning mechanisms remain unclear. Therefore, this study aims to examine the effects of *Coix lacryma-jobi* L. and *Raphanus sativus* L., alongside their active compounds, coixol and sinigrin. **Methods**: Cytotoxicity in 3T3-L1 cells was assessed using the 3-(4,5-dimethylthiazol-2-yl)-2,5-diphenyltetrazolium bromide (MTT) assay. Lipid accumulation was quantified by the Oil Red O (ORO) staining. Immunofluorescence staining was employed to evaluate mitochondrial activity and uncoupling protein 1 (UCP1). Protein and mRNA expressions were analysed using western blot and quantitative real-time polymerase chain reaction (qRT-PCR), respectively. **Results**: In 3T3-L1 adipocytes, ORO staining showed reduced lipid accumulation and droplet size after treatment. qRT-PCR, western blot, and immunostaining revealed that coixol and sinigrin upregulated browning markers (UCP1, PGC-1α, PRDM16) and beige fat genes (*Cd137*, *Cidea*, *Cited*, *Fgf21*, *Tbx1*, *Tmem26*). They also upregulated mitochondrial biogenesis genes (*Cox4*, *Nrf1*, *Tfam*), downregulated lipogenic genes (*Fasn*, *Lpl*, *Srebf1*, *Acaca*), and increased lipolytic (*Atgl*, *Hsl*, *Plin1*) and fatty acid oxidation genes (*Aco1*, *Cpt1*, *Ppara*). Mechanistic studies revealed that fat browning was associated with β3-adrenergic receptor activation and AMPK phosphorylation. **Conclusions**: Overall, coixol and sinigrin promote fat browning and metabolic improvement, highlighting their potential as natural anti-obesity agents.

## 1. Introduction

Obesity is not an issue of appearance but a complex metabolic disease that poses a significant threat to human health [1]. Since the 20th century, rapid urbanization, industrialization, and the westernization of diets have increased high-calorie food consumption and significantly decreased daily physical activity. This shift creates an imbalance between energy intake and expenditure [2]. Consequently, obesity rates have increased rapidly worldwide. The World Health Organization (WHO) recently reported that approximately 16% of adults are obese, which corresponds to about 890 million individuals. When including those who are overweight, the total exceeds 2.5 billion people [3]. Obesity markedly elevates the risk of chronic diseases such as type 2 diabetes, hypertension, nonalcoholic fatty liver disease, and cardiovascular conditions, and it is also linked to higher rates of several cancers. Furthermore, global medical costs related to obesity are projected to reach $3 trillion annually by 2030 [4]. Current obesity treatments primarily focus on restricting energy intake by suppressing appetite or inhibiting fat absorption. However, these strategies often exhibit limited long-term efficacy, and even newer medications, such as glucagon-like peptide 1 (GLP-1) receptor agonists, are associated with adverse effects including nausea, diarrhea, and sarcopenia. Consequently, a fat browning strategy that enhances energy expenditure—specifically by converting white adipose tissue (WAT) into thermogenically active brown-like adipose tissue—has garnered attention as a promising approach to obesity management [5]

Brown adipose tissue (BAT) oxidizes metabolic substrates—such as glucose, fatty acids, and branched-chain amino acids—to generate heat, thereby contributing to systemic metabolic homeostasis. The presence of functional BAT in adults is well established [6]. Increasing attention has focused on targeting BAT as a therapeutic strategy for preventing and treating diabetes, dyslipidemia, and cardiovascular disease [7]. Moreover, the browning of WAT has emerged as a promising strategy in obesity management. While WAT primarily stores energy, it is structurally and functionally distinct from BAT, which is responsible for heat production and energy expenditure. However, some white fat cells can acquire BAT-like properties in response to specific stimuli, a process referred to as WAT browning. This adaptation is associated with enhanced energy expenditure, insulin sensitivity, and lipid metabolism. In mice with high-fat diet-induced obesity, taurine administration increases the expression of browning genes in white fat, thereby suppressing weight gain and improving glycemic control [3]. Additionally, arctiin, a plant-derived lignan, increases uncoupling protein 1 (UCP1) expression in WAT and promotes thermogenesis through adenosine A2A receptor activation, demonstrating anti-obesity efficacy via browning [2]. Furthermore, combined treatment with berberine and evodiamine enhances the expression of UCP1 and peroxisome proliferator-activated receptor gamma coactivator 1α (PGC-1α) in WAT, induces adipocyte browning, and reduces fat mass while improving metabolism in 3T3-L1 cells and a high-fat diet-induced mouse model [4]. Collectively, these findings suggest that functionally reprogramming white fat is a promising strategy for obesity management, extending beyond traditional BAT activation methods.

Kim et al. report that research trends on herbal medicines for obesity in Korea were systematically identified and analyzed [8], revealing that *Taeeumjowi-tang* (TJT) is one of the most used formulas for obesity management. In mice with high-fat diet-induced obesity, TJT reduces the gene expression of sterol regulatory element-binding protein and tumor necrosis factor-α (TNF-α), which play critical roles in lipid and glucose metabolism [9]. Furthermore, a clinical study involving 206 patients with metabolic syndrome and obesity shows that 12 weeks of TJT treatment lowers body mass index and triglyceride levels while improving insulin resistance [10].

*Coix lacryma-jobi* L. (*C. lacryma-jobi*), the primary single-herb component of TJT, belongs to the *Poaceae* (*Gramineae*) family. Traditionally, it has been used to improve spleen function, promote diuresis, relax tendons, and reduce fever [11]. Additionally, it contains a diverse array of compounds, including fatty acids, esters, polysaccharides, sterols, phenols, flavonoids, alkaloids, and triterpenoids [12]. Modern pharmacological studies report that *C. lacryma-jobi* offers multiple health benefits, such as improving liver function, exerting antitumor and anti-obesity effects, regulating the gut microbiota, and reducing inflammation [13,14,15,16,17]. Coixol is distributed throughout various parts of *C. lacryma-jobi*, including roots, stems, leaves, cores, and seed coats. Among them, the roots exhibit the highest concentrations of coixol [18], while the seeds contain relatively lower amounts [19,20,21]. Coixol exhibits pharmacological activities such as mitigating lung injury, reducing inflammation, and improving insulin resistance [22,23,24].

*Raphanus sativus* L. (*R. sativus*), the second most abundant component of TJT, is among the most widely cultivated and consumed vegetables worldwide and belongs to the Brassicaceae (cruciferous) family [25]. Its dried seeds, known as Raphani Semen, have been traditionally used to treat indigestion, bloating, upper abdominal pain, constipation, diarrhea, and dysentery [26]. In herbal medicine, it has also been utilized as a digestive, diuretic, expectorant, anticancer, and anti-inflammatory agent [27]. Over 70 chemical constituents have been identified in *R. sativus*, including flavonoid glycosides, alkaloids, terpenoids, steroids, and oligosaccharides [25]. Contemporary pharmacological studies report that compounds from *R. sativus* have antitumor, anti-inflammatory, antioxidant, antibacterial, antihypertensive, carminative, and digestive properties [28,29,30]. Sinigrin is a naturally occurring aliphatic glucosinolate present in members of the Brassicaceae family, such as broccoli, brussels sprouts, and *R. sativus* [31]. Research shows that sinigrin exerts antibacterial, antioxidant, and anti-inflammatory activities [32]. In line with this trend, many studies are actively being conducted on anti-obesity research through the fat browning effect of active ingredients using natural products [33,34,35,36,37].

Among the various herbal formulas traditionally used for obesity management, TJT has gained attention for its clinical efficacy in improving metabolic parameters [8]. However, while its anti-obesity effects have been reported, the specific components responsible for these benefits and their underlying mechanisms—particularly those related to fat browning—remain unclear. TJT consists primarily of *C. lacryma-jobi* and *R. sativus*, both of which possess anti-obesity and metabolic regulatory properties, likely mediated through their bioactive constituents. In particular, coixol (derived from *C. lacryma-jobi*) and sinigrin (derived from *R. sativus*) have shown potential in modulating inflammation, improving insulin sensitivity, and regulating lipid metabolism [9,10,22,23,27,32]. Nevertheless, their roles in inducing fat browning remain underexplored, warranting further mechanistic investigation. Therefore, this study aims to investigate the fat browning effects and underlying mechanisms of these single herbs (*C. lacryma-jobi* and *R. sativus*) and their active compounds (coixol and sinigrin).

## 2. Results

### 2.1. Extracts of C. lacryma-jobi and R. sativus, Along with Their Active Compounds, Reduce Lipid Accumulation and Lipid Droplet Size

**Figure 1:**
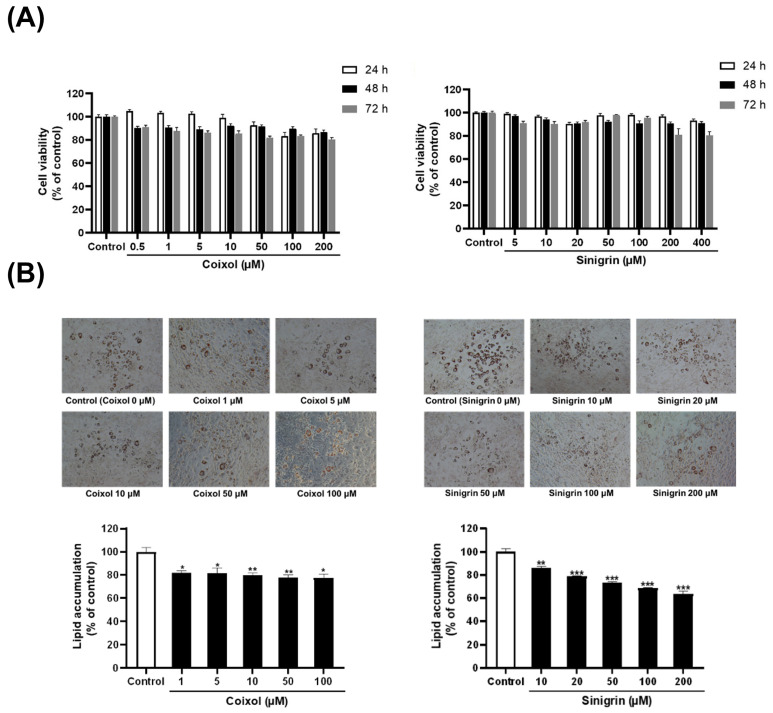
Effects of coixol and sinigrin on cell viability and lipid accumulation in 3T3-L1 adipocytes. (**A**) Cell viability was assessed after 24 h, 48 h, and 72 h of treatment using the MTT assay. Cells were seeded in 96-well plates and incubated for 24 h prior to treatment. Results are expressed as a percentage of the control (0.5% DMSO) (*n* = 6). (**B**) Lipid accumulation was evaluated after 7 days of differentiation. Cells were seeded in 24-well plates, with lipid droplets stained with Oil Red O, extracted using isopropanol, and quantified at 520 nm using a microplate reader. Representative cell images were obtained at 100× magnification. Data are expressed as a percentage of the control (0.5% DMSO) and reported as mean ± standard error of the mean (SEM) from triplicates. * *p* < 0.05, ** *p* < 0.01, and *** *p* < 0.001 vs. control.

Cell viability of single herbs and their active compounds was assessed using the 3-(4,5-dimethylthiazol-2-yl)-2,5-diphenyltetrazolium bromide (MTT) assay was performed in 3T3-L1 preadipocytes. Figure S1 shows that extracts of *C. lacryma-jobi* (CLE) and *R. sativus* (RSE) were incubated for 24, 48, and 72 h at graded concentrations (1, 5, 10, 25, 50, 75, and 100 μg/mL). Both extracts exhibited good biocompatibility with 3T3-L1 adipocytes, with cell viability remaining above 100% across all concentrations and time points. Furthermore, the effects of their active compounds, such as coixol and sinigrin, on cell viability were evaluated. After 72 h of incubation at maximum concentrations of 200 μM and 400 μM, respectively, coixol and sinigrin maintained approximately 80% cell viability (Figure 1A). Based on these findings, subsequent experiments employed different doses of the extracts (1, 5, 10, 25, 50, 75, and 100 μg/mL) and coixol and sinigrin (0.5, 1, 5, 10, 20, 50, 100, 200, and 400 μM), none of which inhibited cell proliferation or induced cytotoxicity compared to that of the control.

Next, we evaluated the effects of single herbs and their active compounds on lipid accumulation, 3T3-L1 preadipocytes were differentiated for 7 days, fixed in 10% formalin, and stained with Oil Red O. Treatment with varying extract concentrations (1–100 μg/mL) showed that CLE and RSE decreased lipid droplet size while increasing the number of small lipid droplets compared to those of the control group (Figure S1). Lipid-bound Oil Red O was then eluted with 100% isopropanol and quantified. Consequently, all extracts significantly reduced lipid accumulation. Furthermore, coixol and sinigrin inhibited lipid accumulation in a concentration-dependent manner within the treated range (1–200 μM; Figure 1B).

### 2.2. C. lacryma-jobi and R. sativus, Along with Their Active Compounds, Contribute to Fat Browning in 3T3-L1 Adipocytes

**Figure 2:**
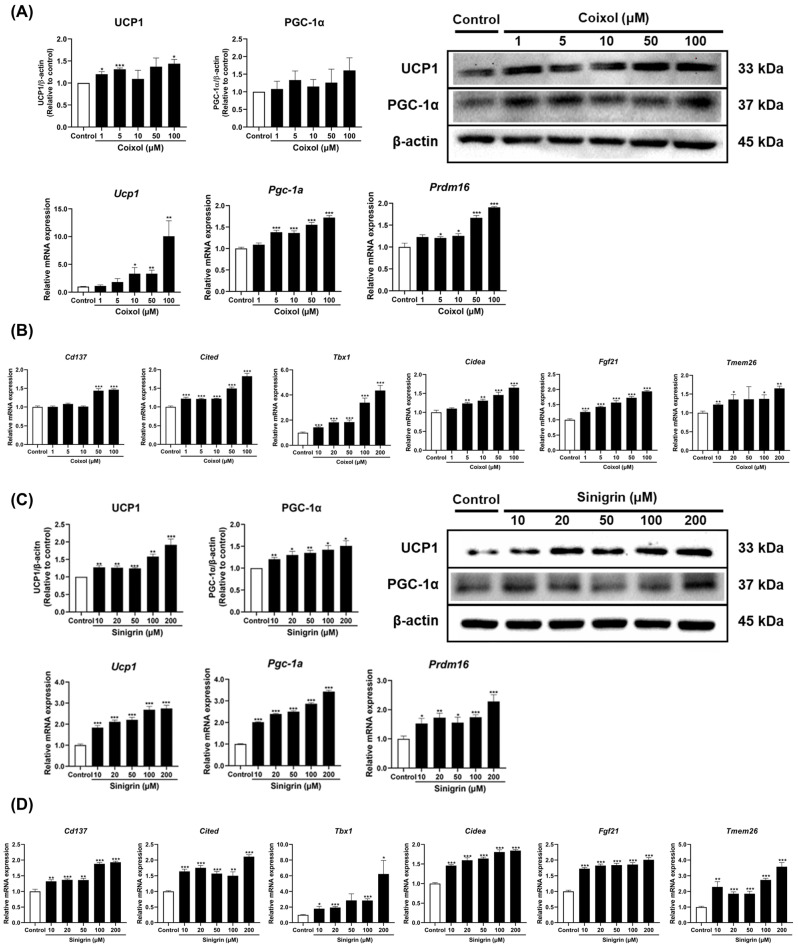
Effects of coixol (**A**,**B**) and sinigrin (**C**,**D**) on the expression of thermogenic and beige fat-specific markers in 3T3-L1 adipocytes. Target gene mRNA levels were normalized to *Gapdh* using the 2–ΔΔCt method (*n* = 6). *Gapdh* was used as the housekeeping gene, while β-actin served as the loading control for protein expression (*n* = 3). Results are presented as mean ± standard error of the mean (SEM). * *p* < 0.05, ** *p* < 0.01, and *** *p* < 0.001 vs. control.

As previously described, UCP1 is mainly expressed in BAT and beige adipocytes, where it drives thermogenesis and lipid mobilization in WAT. Elevated PGC-1α and PRDM16 acted as reciprocal coactivators, facilitating fat browning [38]. Additionally, markers that distinguish beige adipocytes have been identified [39]. To investigate whether the extracts and their active compounds reduced lipid accumulation through a fat browning effect, quantitative real-time polymerase chain reaction (qRT-PCR) and western blot analyses were performed to assess relevant targets at the mRNA and protein levels, respectively. First, extracts of the single herbs dose-dependently upregulated the expression of thermogenic regulators *Ucp1*, *Pgc-1a*, and *Prdm16*, which are key factors of fat browning. They also significantly increased the expression of beige-specific markers such as *Cd137*, *Cidea*, *Cited*, and *Fgf21*, indicating a fat browning effect (Figure S2). Moreover, coixol and sinigrin upregulated thermogenic gene expression and significantly increased UCP1 and PGC-1α protein levels (Figure 2A,C). Both compounds also significantly increased the expression of beige-specific marker genes, including *Cd137*, *Cidea*, *Cited*, *Fgf21*, *Tbx1*, and *Tmem26* in 3T3-L1 adipocytes (Figure 2B,D). These findings indicate that the single-herb extracts and their active compounds promote the browning of 3T3-L1 adipocytes by activating thermogenic pathways.

### 2.3. C. lacryma-jobi and R. sativus, Along with Their Active Compounds, Enhance Mitochondrial Biogenesis in 3T3-L1 Adipocytes

**Figure 3:**
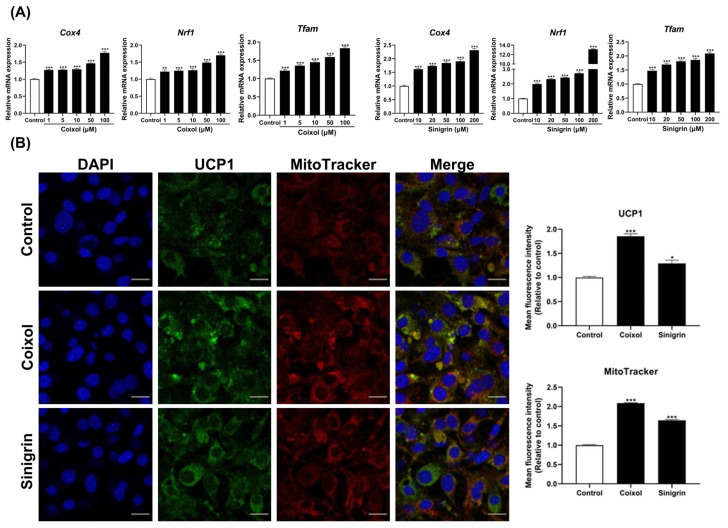
Effects of coixol and sinigrin on mitochondrial biogenesis in 3T3-L1 adipocytes. (**A**) mRNA expression of mitochondrial biogenesis markers was evaluated using qRT-PCR. *Gapdh* was used as the housekeeping gene, and target gene expression was normalized using the 2−ΔΔCt method. Results are presented as mean ± standard error of the mean (SEM) (*n* = 6). (**B**) Effects of their active compounds on intracellular mitochondrial biogenesis, with UCP1 activation evaluated using immunofluorescence staining (*n* = 3). UCP1 protein localization was visualized using FITC-conjugated antibody (UCP1-FITC, green), DAPI (nuclei, blue), and MitoTracker Red (mitochondria, red). Images were obtained at 60× magnification (scale bar = 10 μm). * *p* < 0.05, ** *p* < 0.01, and *** *p* < 0.001 vs. control.

Mitochondria fulfill several critical functions in adipose tissue, including fatty acid oxidation, glucose oxidation, adipogenesis, lipogenesis, and lipolysis [40,41]. To further evaluate the effects of single-herb extracts and their active compounds on mitochondrial biogenesis, the expression of key biogenesis-related genes (*Cox4*, *Nrf1*, *Tfam*) was determined. The extracts (Figure S3A) and their active compounds (Figure 3A) significantly upregulated the expression of mitochondrial marker genes, consistent with the observed induction of fat browning in 3T3-L1 adipocytes. Immunofluorescence assays using MitoTracker Red and UCP1-FITC were performed to determine whether single-herb extracts and their active compounds enhanced mitochondrial biogenesis and UCP1 expression in 3T3-L1 adipocytes. Immunofluorescence staining revealed that treatment with the highest concentrations of extracts (Figure S3B) and active compounds (Figure 3B) increased mitochondrial abundance and UCP1 expression compared to that of the control group. Collectively, these findings suggest that single-herb extracts and their active compounds may promote fat browning by enhancing mitochondrial biogenesis. Therefore, future experiments should investigate the specific effects of these active compounds.

### 2.4. Coixol and Sinigrin Improved Lipid Metabolism in 3T3-L1 Adipocytes

**Figure 4:**
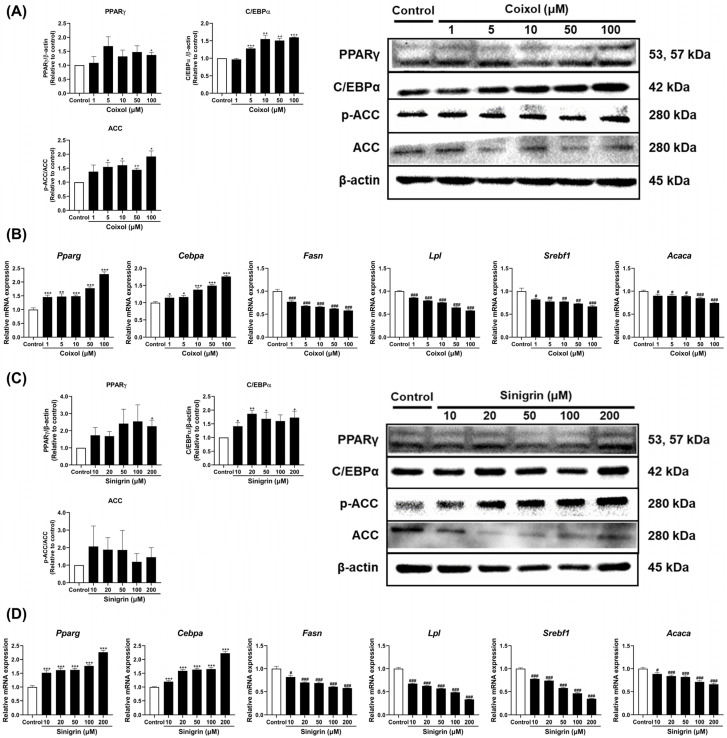
Effects of coixol (**A**,**B**) and sinigrin (**C**,**D**) on the expression of adipogenic and lipogenic markers in 3T3-L1 adipocytes. Target gene mRNA expression was analyzed using qRT-PCR and normalized to *Gapdh* using the 2–ΔΔCt method (*n* = 6). *Gapdh* was used as the housekeeping gene, while β-actin served as the loading control (*n* = 3). Data are expressed as mean ± standard error of the mean (SEM). * *p* < 0.05, ** *p* < 0.01, and *** *p* < 0.001 vs. control (increase); # *p* < 0.05, ## *p* < 0.01, and ### *p* < 0.001 vs. control (decrease).

The effects of coixol and sinigrin on adipogenesis, lipogenesis, and lipid metabolism were investigated at gene and protein levels. Both coixol and sinigrin exhibited the highest protein expression levels of Peroxisome proliferator-activated receptor γ (PPARγ), CCAAT/enhancer binding protein α (C/EBPα) at their maximum concentrations (Figure 4A,C), and consistently, treatment with coixol and sinigrin dose-dependently increased *Pparg* and *Cebpa* mRNA expression (Figure 4B,D). Conversely, these active compounds significantly decreased the expression of lipogenic genes, such as *Fasn*, *Lpl*, *Srebf1*, and *Acaca*, which contribute to fat synthesis in 3T3-L1 adipocytes. Similar to the *Acaca* gene expression, treatment with coixol and sinigrin suppressed acetyl-CoA carboxylase (ACC) protein levels while increasing phosphorylated ACC (p-ACC; Figure 4A,C). These findings suggest that coixol and sinigrin treatment promotes beige adipocyte differentiation, reduces lipogenesis, and enhances fat browning.

### 2.5. Coixol and Sinigrin Promoted Lipid Catabolism in 3T3-L1 Adipocytes

**Figure 5:**
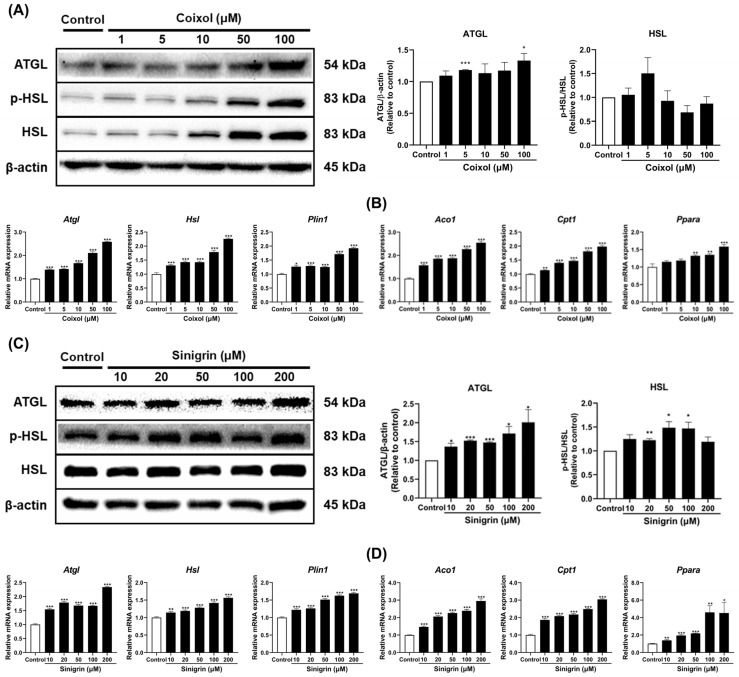
Effects of coixol (**A**,**B**) and sinigrin (**C**,**D**) on the expression of lipolytic and β-oxidation markers in 3T3-L1 adipocytes. Target gene mRNA expression was analyzed using qRT-PCR and normalized to *Gapdh* using the 2–ΔΔCt method (*n* = 6). *Gapdh* was used as the housekeeping gene and β-actin as the loading control (*n* = 3). Data are presented as mean ± standard error of the mean (SEM). * *p* < 0.05, ** *p* < 0.01, and *** *p* < 0.001 vs. control.

Lipolysis is a catabolic process in which triacylglycerols stored in adipocytes are broken down into free fatty acids and glycerol [42]. Adipose triglyceride lipase (ATGL) initiates lipolysis by hydrolyzing triacylglycerols into diacylglycerols and activating hormone-sensitive lipase (HSL), which subsequently hydrolyzes diacylglycerols into monoacylglycerols [43]. Together, ATGL and HSL account for approximately 95% of total lipolytic activity. Perilipin 1 (PLIN1) regulates these enzymes by controlling their access to lipid droplets [44]. Figure 5 shows that coixol and sinigrin significantly increased the expression of lipolysis-related genes (*Atgl*, *Hsl*, *Plin1*), consistent with their corresponding protein levels (Figure 5A,C). Additionally, both compounds increased the expression of fatty acid oxidation genes (*Aco1*, *Cpt1*, *Ppara*) (Figure 5B,D). Overall, these findings confirm that coixol and sinigrin modulate lipid metabolism and catabolism, thereby promoting fat browning in 3T3-L1 adipocytes.

### 2.6. Coixol and Sinigrin Indicate Variations in Fat Browning via β3-AR, AMPK Signaling Pathways in 3T3-L1 Adipocytes

**Figure 6:**
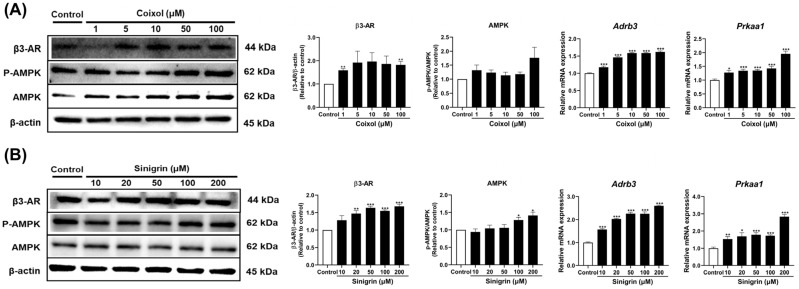
Effects of (**A**) coixol and (**B**) sinigrin on the expression of fat browning-associated signaling pathway targets in 3T3-L1 adipocytes. Target gene mRNA expression was analyzed using qRT-PCR and normalized to *Gapdh* using the 2^−ΔΔCt^ method (*n* = 6). *Gapdh* was used as the housekeeping gene for mRNA analysis, and β-actin as the loading control for protein analysis (*n* = 3). Data are presented as mean ± standard error of the mean (SEM). * *p* < 0.05, ** *p* < 0.01, and *** *p* < 0.001 vs. control.

Beta 3 adrenergic receptor (β3-AR) plays a central role in regulating lipolysis and thermogenesis [45]. To elucidate the molecular mechanism underlying the browning effects of coixol (Figure 6A) and sinigrin (Figure 6B), we examined β3-AR expression. The gene and protein expression of β3-AR (gene name: *Adrb3*) increased following treatment with both compounds.

Adenosine monophosphate-activated protein kinase (AMPK) regulates the breakdown and synthesis of glucose, lipids, and cholesterol in the liver, muscle, and adipose tissues, playing a key role in energy metabolism [46]. It is also involved in the differentiation and activation of brown and beige adipocytes [47]. To investigate the mechanism underlying coixol- (Figure 6A) and sinigrin-mediated (Figure 6B) UCP1 activation via the AMPK signaling pathway, additional studies were conducted in 3T3-L1 adipocytes. Treatment with coixol and sinigrin increased the expression of AMPK (gene name: *Prkaa1*), the predominant isoform in adipose tissue. Furthermore, sinigrin alone significantly upregulated AMPK phosphorylation.

As shown in Figure S4A–D, co-treatment with the β3-AR agonist BRL 37,344 or the AMPK activator AICAR further enhanced the expression of β3-AR and AMPK, whereas treatment with the β3-AR antagonist L-748,337 or the AMPK inhibitor of dorsomorphin abolishes these increases. A similar pattern was also observed for UCP1 expression, confirming that thermogenic effects of coixol and sinigrin are dependent on β3-AR and AMPK pathway activation.

## 3. Discussion

Obesity is a major public health concern closely associated with type 2 diabetes, insulin resistance, hypertension, and cardiovascular disease [48]. It arises from a complex interaction of genetic, environmental, lifestyle, and psychological factors. The prevalence of obesity has rapidly increased in modern society, driven by higher energy intake from calorie-dense diets and decreased energy expenditure due to sedentary lifestyles [49]. Overeating, resulting from disruptions in hormones and neurotransmitters that regulate appetite, is a key contributor to obesity. The hypothalamus contains a feeding center in its ventrolateral region and a satiety center in its ventromedial region. Overeating occurs when satiety signaling is impaired and the feeding center is enhanced [50]. Most anti-obesity drugs developed so far act by suppressing energy intake. However, many have been withdrawn due to serious adverse effects, such as cardiovascular issues, increased risk of suicidal behavior, and a higher incidence of certain cancers. Additionally, sustaining weight loss through dietary restriction is challenging, as the body retains an obesogenic memory that resists weight reduction, increasing the risk of weight regain, or the “yo-yo” effect [51]. Fat browning, the conversion of white adipocytes into metabolically active beige adipocytes that function similarly to brown adipocytes, enhances energy expenditure [52]. This process increases basal metabolic rate, supports long-term weight management, and promotes overall energy expenditure, thereby addressing the underlying cause of fat accumulation [53]. Additionally, fat browning enhances glucose and lipid metabolism while improving cardiometabolic homeostasis [54].

In this study, the single herbs (*C. lacryma-jobi* and *R. sativus*) and their active compounds (coixol and sinigrin) were evaluated for their effects on fat browning in 3T3-L1 adipocytes, with concentrations selected based on non-cytotoxic ranges established through cell viability assay and previously reported effective doses in adipocyte models to ensure physiological relevance of the experimental design. The results showed that all single herbs and their active compounds reduced lipid accumulation and decreased lipid droplet size. These findings suggest that they may mitigate obesity progression by suppressing adipocyte hyperplasia and hypertrophy. Thermogenesis employs stored triglycerides for energy, thereby reducing lipid accumulation in adipocytes. As mentioned earlier, UCP1 is essential for thermogenesis, facilitating proton leakage across the inner mitochondrial membrane to generate heat rather than ATP [6]. PGC-1α upregulates multiple genes involved in mitochondrial biogenesis and lipid metabolism, promoting thermogenesis through UCP1 induction [55]. Additionally, PRDM16, a cell-autonomous regulator of thermogenesis, enhances UCP1 expression and other thermogenesis-related genes [56]. The single herbs and their active compounds promote thermogenesis by upregulating UCP1, PGC-1α, and PRDM16. They also significantly upregulate the expression of beige adipocyte-specific markers, including *Cd137*, *Cidea*, *Cited*, *Fgf21*, *Tbx1*, and *Tmem26* [57]. Immunofluorescence analysis further revealed that treatment with these herbs and their active compounds increased UCP1 expression and mitochondrial content. These findings align with the upregulation of mitochondrial biogenesis genes, such as *Cox4*, *Nrf1*, and *Tfam*. Overall, *C. lacryma-jobi* and *R. sativus*, along with their active compounds (coixol and sinigrin), induce fat browning by enhancing thermogenesis, upregulating beige adipocyte-specific markers, and promoting the expression of mitochondrial biogenesis-related genes.

To investigate the mechanisms underlying lipid metabolism regulation through fat browning, the effects of coixol and sinigrin on adipogenic and lipogenic markers were evaluated. Several adipogenic transcription factors were identified, with PPARγ serving as the principal regulator of adipocyte differentiation and gene expression [58]. PPARγ, a member of the nuclear hormone receptor superfamily, is essential for adipocyte development both in vitro and in vivo [59]. Additionally, C/EBPα is a key transcription factor that promotes the differentiation of preadipocytes into both white and brown adipocytes [60]. Interestingly, although PPARγ and C/EBPα are classically associated with adipogenesis, recent studies have demonstrated that PPARγ also plays a pivotal role with PRDM16 in induction and maintenance of beige adipocytes [61]. Lipogenesis refers to the cellular processes of exogenous fatty acids absorbing and synthesizing fatty acids from alternative carbon sources to maintain lipid homeostasis [62,63]. *Fasn* encodes a key enzyme in fatty acid synthesis and directly contributes to lipid accumulation in adipose tissue [64]. *Lpl* is a key enzyme that facilitates adipocyte uptake of circulating lipids, while *Srebf1* is a transcription factor that regulates fatty acid and triglyceride synthesis [65,66]. *Acaca* encodes an enzyme that initiates fatty acid synthesis by producing malonyl-CoA, marking the early state of the pathway [67]. Coixol and sinigrin promote beige adipocyte differentiation by increasing PPARγ and C/EBPα levels. Additionally, the active compounds downregulated the expression of lipogenic genes, including *Fasn*, *Lpl*, *Srebf1*, and *Acaca*. They reduce ACC protein levels while increasing p-ACC, indicating suppressed fatty acid synthesis and enhanced energy expenditure. In summary, coixol and sinigrin promote beige adipocyte formation by stimulating adipogenesis and inhibiting lipogenesis.

Next, the effects of coixol and sinigrin on lipid catabolism were evaluated. Lipolysis involves the breakdown of triglycerides into free fatty acids and glycerol in adipocytes. Activation of this pathway reduces lipid accumulation in white adipocytes and promotes beige adipocyte induction [68]. ATGL, HSL, and *Plin1* are key regulators in lipolysis. ATGL initiates lipolysis by hydrolyzing triglycerides into diacylglycerol and free fatty acids [69]. Subsequently, HSL cleaves diacylglycerol into monoacylglycerol and free fatty acids, serving as the key enzyme in the second step of lipolysis. Its activity is enhanced through phosphorylation, regulated by hormonal signals [70]. *Plin1* encodes a protein that coats the surface of lipid droplets and inhibits ATGL activation under basal conditions. Upon phosphorylation, Plin1 promotes the translocation of phosphorylated HSL (p-HSL) to the lipid droplet surface, thereby activating lipolysis [71]. Free fatty acid oxidation is a crucial metabolic pathway that utilizes fatty acids as an energy source. *Aco1* catalyzes the first step of fatty acid β-oxidation in peroxisomes, cleaving fatty acids into 2-carbon units to produce acetyl-CoA. This activity regulates peroxisomal fatty acid oxidation and is crucial for energy production and lipid homeostasis [72]. *Cpt1*, located in the outer mitochondrial membrane, regulates the balance between fatty acid synthesis and oxidation by conjugating long-chain fatty acids to carnitine for mitochondrial transport [73]. *Ppara* is a transcription factor regulating the expression of genes involved in fatty acid metabolism, directly upregulating *Aco1* and *Cpt1*. This enzyme promotes fatty acid oxidation in both peroxisomes and mitochondria [74]. Activating these pathways in adipocytes induces functional changes in adipose tissue, offering a potential therapeutic strategy for obesity and metabolic diseases. Coixol and sinigrin increased the gene and protein expression of ATGL, HSL, and p-HSL and upregulated *Plin1* expression. Additionally, the compounds enhanced the expression of *Aco1*, *Cpt1*, and *Ppara*. These findings indicate that coixol and sinigrin facilitate energy expenditure and metabolic health by activating lipolysis and free fatty acid oxidation, key mechanisms of fat browning.

Obesity is attributed to disruptions in energy metabolism, inhibiting the efficient utilization of expended energy. During overnutrition, the thyroid hormone thyroxine (T4) is converted to triiodothyronine (T3) in peripheral tissues, leading to increased norepinephrine, a key neurotransmitter of the sympathetic nervous system [75]. Norepinephrine then binds to beta-adrenergic receptors on adipocyte membranes, initiating energy expenditure pathways [76]. The increase in intracellular cAMP levels activates protein kinase A (PKA), indirectly activating AMPK [77]. Dysfunction of this pathway reduces energy expenditure, ultimately contributing to obesity. To evaluate the molecular mechanisms underlying the fat browning effects of coixol and sinigrin, targets in the β3-AR and AMPK signaling pathways were investigated. Coixol increased the mRNA expression of *Adrb3* and *Prkaa1*, while sinigrin treatment similarly enhanced AMPK phosphorylation (Figure 6A,B). Moreover, in Figure S4A–D, co-treatment with the β3-AR agonist (BRL 37,344) or the AMPK activator (AICAR) further amplified β3-AR, AMPK, and UCP1 expression. In contrast, treatment with the β3-AR antagonist (L-748,337) or the AMPK inhibitor (Dorsomorphin) abolished these effects. These results confirm that β3-AR and AMPK activation and indispensable mediators of the thermogenic response induced by coixol and sinigrin. Collectively, these findings demonstrated that coixol and sinigrin promote fat browning by activating UCP1 through β3-AR- and AMPK-dependent signaling in 3T3-L1 adipocytes.

Studies show various pharmacological effects of the individual herbs (*C. lacryma-jobi* and *R. sativus*) and their active compounds (coixol and sinigrin). However, this study is the first to clarify how these herbs and compounds modulate gene expression and protein levels associated with fat browning, highlighting their potential for obesity management. Furthermore, this study demonstrated the molecular mechanisms underlying the fat browning effects of these compounds, particularly with the activation of AMPK and β3-AR signaling pathways as central mediators. Similar to our finding, several natural compounds—such as D-mannitol, which induces a brown fat-like phenotype through β3-AR dependent mechanisms, and ginsenoside Rb1, which promotes thermogenesis and lipolysis via β3-AR receptor activation in both 3T3-L1 adipocytes and C57BL6/J mice—have been reported to regulate adipocyte browning through AMPK and β3-AR [78,79]. These mechanistic parallels further support the role of coixol and sinigrin as potential thermogenic agents that act through comparable molecular pathways. Given that coixol and sinigrin enhance thermogenic and lipolytic signaling in 3T3-L1 adipocytes, it is plausible that these compounds may also exert anti-obesity effects in vivo by improving metabolic homeostasis. Nevertheless, as this study was conducted solely in vitro, further in vivo validation using diet-induced obesity models will be essential to confirm these mechanistic insights. These compounds present strong therapeutic potential as anti-obesity agents, facilitating adipose tissue browning and offer the potential for developing combination formulations by analyzing their interactions and synergistic effects. This foundation supports further research in animal models and clinical studies to address the limitations of existing drugs. Furthermore, these findings provide valuable insights for developing functional foods or anti-obesity supplements derived from traditional herbal medicines with enhanced safety profiles. In summary, this study provides scientific validation of these herbal medicines and their active compounds, establishing a robust data foundation for developing modern anti-obesity therapeutics.

## 4. Materials and Methods

### 4.1. Chemicals and Reagents

Dried seeds of *C. lacryma-jobi* (14.3 kg) were purchased from a local market in July 2024. Dried ripe seeds of *R. sativus* (2.2 kg) were generously provided by Professor Young-Sam Keum, College of Pharmacy, Dongguk University (Goyang, Republic of Korea). Voucher specimens for *C. lacryma-jobi* (DGU-915) and *R. sativus* (DGU-916) were deposited at the Integrated Research Institute for Drug Development, Dongguk University in July 2024. Each seed material was pulverized into a fine powder and extracted three times with 70% EtOH (16 L × 3, 99 min each) by ultrasonication at room temperature. The combined extracts were filtered through cotton wool and then concentrated under reduced pressure at 40 °C using a rotary evaporator to yield the crude extracts, CLE (289.33 g) and RSE (272.8 g), respectively. The extraction procedures for both seeds were identical and followed previously reported methods [80,81]. Coixol and sinigrin were purchased from ChemFaces Biochemical (Wuhan, China). The 3T3-L1 adipocyte cell line was obtained from the Korean Cell Line Bank (Seoul, Republic of Korea). Dulbecco’s Modified Eagle Medium (DMEM; high glucose), fetal bovine serum (FBS), newborn calf serum (NCS), penicillin–streptomycin solution (P-S), and 4′,6-diamidino-2-phenylindole (DAPI) were purchased from Thermo Fisher Scientific (Waltham, MA, USA). Dexamethasone (DEX), insulin, 5-aminoimidazole-4-carboxamide ribonucleotide (AICAR), dorsomorphin, phosphate-buffered saline (PBS), 10% formalin, and Oil Red O were purchased from Sigma-Aldrich (St. Louis, MO, USA). DMSO and MTT were purchased from Glentham Life Sciences (Corsham, UK). Isopropanol, 3-isobutyl-1-methylxanthine (IBMX), goat anti-rabbit IgG secondary antibody, and goat anti-mouse IgG secondary antibody were obtained from Merck (Union County, NJ, USA). Rabbit monoclonal or polyclonal antibodies against ATGL (2439, 1:1000), PKA (5842, 1:1000), PLIN (9349, 1:1000), PPARγ (2435, 1:1000), C/EBPα (8178, 1:1000), HSL (18381, 1:1000), p-HSL (4139, 1:1000), AMPK (2532, 1:1000), phospho-AMPK (2531, 1:500), ACC (3662, 1:1000), p-ACC (3661, 1:500), and β-actin (4967, 1:1000) were purchased from Cell Signaling Technology (Danvers, MA, USA). Mouse monoclonal antibodies against UCP1 (sc-293418, 1:500), PGC-1α (sc-518025, 1:500), and β3-AR (sc-515763, 1:500) were purchased from Santa Cruz Biotechnology (Santa Cruz, CA, USA). Immun-Blot^®^ polyvinylidene fluoride (PVDF) membrane, phosphatase inhibitor, protease inhibitor, skim milk, 10% Tween 20 solution, 2-mercaptoethanol (2-βME), 4× Laemmli sample buffer, and iQ™ SYBR^®^ Green Supermix were obtained from Bio-Rad (Hercules, CA, USA). PCR-grade RNase-free water was purchased from Jena Bioscience (Munich, Germany). BRL 37,344 and L-748,337 were obtained from Tocris Bioscience (Bristol, UK). NucleoZOL was obtained from Macherey-Nagel (Düren, Germany), while the ReverTra Ace^®^ qPCR RT Kit was purchased from TOYOBO (Osaka, Japan).

### 4.2. Cell Culture and Differentiation

The 3T3-L1 preadipocytes were cultured for 2 days in DMEM supplemented with 10% NCS and 1% P-S at 37 °C in a humidified 5% CO_2_ incubator. To induce growth arrest, the cells were maintained in the same medium. After reaching full confluence, the culture medium was replaced with differentiation initiation medium (DIM) comprising DMEM supplemented with 10% FBS, 0.5 mM IBMX, 10 μg/mL insulin, and 1 μM DEX. After 3 days, the differentiation medium was replaced with differentiation progression medium (DPM), comprising DMEM supplemented with 10% FBS and 10 μg/mL insulin. After 2 days, the cells were switched to fresh DPM (until day 7). During the differentiation period, 3T3-L1 adipocytes were treated with various concentrations of single herb extracts (1–100 μg/mL) or their active compounds (1–200 μM).

### 4.3. Cell Viability Assay

Cell viability was assessed using the MTT assay. In metabolically active cells, mitochondrial NADPH-dependent oxidoreductases and dehydrogenases reduced the tetrazolium dye to insoluble purple formazan crystals [82]. The 3T3-L1 preadipocytes were seeded overnight in 96-well plates at a density of 1 × 10^4^ cells per well in DMEM supplemented with 10% NCS and 1% P-S. After reaching 80–90% confluence, the cells were treated with various concentrations of CLE (1–100 µg/mL), RSE (1–100 µg/mL), coixol (0.5–200 μM), or sinigrin (5–400 μM). Treatments were applied for 24 h, 48 h, or 72 h. Cells were treated with 0.5% DMSO served as the control group. At each designated time point, 20 μL of 5 mg/mL MTT solution was added to each well and incubated for 2 h. Subsequently, the supernatant was removed, and 100 μL of DMSO was added to dissolve the formazan crystals for 10 min. Cell viability was measured using the xMark™ microplate absorbance spectrophotometer (Bio-Rad, Hercules, CA, USA) at an optical density (OD) of 540 nm. Results were expressed as a percentage of control values based on six independent experiments.

### 4.4. Oil Red O Staining and Quantification

Lipid accumulation was evaluated using Oil Red O staining. Differentiated 3T3-L1 adipocytes were seeded at a density of 1 × 10^5^ cells per well in 24-well plates, washed twice with PBS, and fixed with 10% formalin for 30 min at room temperature. After fixation, the formalin was removed, and the cells rinsed twice with deionized water (DW). Subsequently, the cells were stained with Oil Red O working solution for 30 min at room temperature (25 °C). The Oil Red O working solution was removed, and the cells were washed three times with DW. Microscopic images were captured to visualize stained lipid droplets in the differentiated 3T3-L1 adipocytes. Subsequently, 100% isopropanol was added, and the plates were gently shaken for 30 min at room temperature (25 °C) to extract the dye. The supernatant was transferred to 96-well plates, and absorbance was measured at 520 nm using a microplate reader (Bio-Rad, Hercules, CA, USA).

### 4.5. Western Blot Analysis

Differentiated 3T3-L1 adipocytes were seeded at 1 × 10^6^ cells per well in 6-well plates and washed with ice-cold PBS. The cells were collected into pre-chilled 1.5 mL microcentrifuge tubes (Eppendorf, Hamburg, Germany) and centrifuged at 14,000 rpm for 5 min. Subsequently, the supernatant was discarded, with the resulting cell pellet lysed on ice for 30 min in 100 μL of lysis buffer supplemented with protease and phosphatase inhibitors. The lysates were centrifuged at 14,000× *g* for 20 min, with protein concentrations determined using the Bradford assay. Equal amounts of protein (40 µg) from each sample were adjusted to a total volume of 20 µL with sample buffer and DW. The samples were heated at 100 °C for 10 min in a heating block and centrifuged at 14,000× *g* for 3 min. The proteins were then loaded onto a 10% separating gel and resolved using sodium dodecyl sulfate–polyacrylamide gel electrophoresis (SDS-PAGE) for 1 h. SDS-PAGE-separated proteins were electrotransferred onto an Immun-Blot^®^ PVDF membrane for 1 h. The membrane was blocked with 5% skim milk at room temperature for 1 h on an orbital shaker. After blocking, the membrane was washed five times for 5 min each with Tris-buffered saline containing 0.1% Tween-20 (TBS-T) at room temperature (25 °C), followed by incubation with primary antibodies overnight at 4 °C. After three washes with TBS-T buffer for 5 min, the membrane was incubated with anti-rabbit or anti-mouse IgG secondary antibodies (1:4000, prepared in TBS-T containing 5% skim milk) for 1 h at room temperature (25 °C). Secondary antibody binding was detected using a ChemiDoc™ XRS+ imaging system (Bio-Rad, Hercules, CA, USA), and band intensities were quantified using the Image Lab software 3.0 (Bio-Rad, Hercules, CA, USA).

### 4.6. Quantitative Real-Time Polymerase Chain Reaction

qRT-PCR was conducted to evaluate the mRNA expression of genes associated with adipocyte browning. Differentiated 3T3-L1 adipocytes (1 × 10^6^ cells/well, 6-well plate) were lysed in 500 μL of NucleoZOL (Macherey-Nagel, Düren, Germany). An equal volume (500 μL) of PCR-grade RNase-free water was added, and the lysates were centrifuged at 12,000× *g* for 15 min. The resulting supernatant (1 mL) was transferred to a new tube, and RNA was precipitated by adding 1 mL of isopropanol. The samples were incubated at room temperature (25 °C) for 10 min and centrifuged at 12,000× *g* for 10 min. The resulting pellets were washed with 500 μL of 75% EtOH, followed by centrifugation at 8000× *g* for 3 min. After removing the supernatant, total RNA was resuspended in PCR-grade RNase-free water. RNA purity and concentration were determined using a Nanodrop spectrophotometer (Thermo Scientific, Wilmington, DE, USA). Subsequently, mRNA was purified from total RNA using the NucleoTrap^®^ mRNA Mini purification Kit (Macherey-Nagel, Düren, Germany). Full-length complementary DNAs were synthesized from mRNA using the ReverTra Ace^®^ qPCR RT Kit (Toyobo, Osaka, Japan) following the instructions of the manufacturer. qRT-PCR was performed on a CFX384 Touch Real-Time PCR Detection System (Bio-Rad, Hercules, CA, USA) using iQ™ SYBR^®^ Green Supermix to quantify transcript levels of target genes. mRNA expression levels were normalized to *Gapdh* using the 2-ΔΔCt method. Table 1 lists the primer sequences used in this study.

### 4.7. Immunofluorescence Staining

Immunofluorescence analysis was performed to evaluate the effects of individual herbs and their active compounds on UCP1 expression and mitochondrial biogenesis in 3T3-L1 adipocytes. Sterilized coverslips were placed into each well of a 24-well plate, and the cells were seeded onto the coverslips and differentiated for 7 days. For mitochondrial staining, the cells were incubated in medium containing 50 nM MitoTracker^®^ Red CMXRos (Thermo Fisher Scientific, Waltham, MA, USA) for 30 min. The cells were rinsed three times with PBS and fixed with 10% formalin for 15 min at room temperature (25 °C). Subsequently, they were washed three additional times with PBS and incubated in blocking buffer (5% BSA, 0.1% Triton^®^ X-100 in PBS) for 1 h at 4 °C. The cells were washed with blocking buffer and incubated overnight at 4 °C with an FITC-conjugated primary antibody against UCP1 (1:500). They were washed three times with PBS for 5 min each, stained with DAPI (10 mg/mL, 1:1000) for 1 min to visualize nuclei, and washed again with PBS. Fluorescence images were acquired using a Nikon C1 confocal laser scanning microscope and analyzed with EZ-C1 software 3.9 (Nikon, Tokyo, Japan) and quantitative fluorescence intensity was assessed using ImageJ version 1.54 (National Institute of Health, Bethesda, MD, USA) [83].

### 4.8. Statistical Analysis

All data are presented as mean ± standard error of the mean (SEM). Western blot and immunofluorescence experiments were conducted in triplicate, while all other experiments were performed with six replicates. Differences between control and treatment groups at each concentration were analyzed using Student’s *t*-test. Statistically significant difference was defined as *p* < 0.05.

## 5. Conclusions

A limited understanding of the mechanisms underlying traditional herbal medicine remains a significant barrier to advancing research and development in modern healthcare. Clarifying these mechanisms of action is clinically crucial. Furthermore, current obesity treatments primarily focus on reducing energy intake by suppressing appetite or inhibiting absorption. In contrast, fat browning was examined as a promising mechanism that enhances energy expenditure. This study is the first to demonstrate that the single herbs (*C. lacryma-jobi* and *R. sativus*) and their active compounds (coixol and sinigrin) significantly reduce lipid accumulation in 3T3-L1 adipocytes. Additionally, these treatments upregulated UCP1, PGC-1α, and PRDM16, while increasing the expression of beige fat-specific marker genes. These effects are associated with the induction of beige adipocytes, enhancing mitochondrial biogenesis. The bioactive compounds positively regulate markers involved in lipid metabolism and catabolism. A mechanistic study using 3T3-L1 cells reveals a novel role for these compounds in inducing UCP1-dependent thermogenesis via activation of the β3-AR/AMPK signaling pathway. Although this work was limited to an in vitro model, these findings provide valuable mechanistic insight and highlight the need for further in vivo validation to confirm their therapeutic potential. In addition, the complementary actions of coixol and sinigrin suggest potential applicability in developing combined nutraceutical formulations that promote adipose tissue browning and metabolic health. These findings suggest that coixol and sinigrin are promising therapeutic candidates for obesity management and offer a valuable foundation for future in vivo studies, serving as crucial in vitro baseline data for developing safe and effective anti-obesity therapies.

## Figures and Tables

**Table 1:** The primer sequence used for quantitative RT-PCR.

Gene	Forward	Reverse
*Acaca*	GGGAACATCCCCACGCTAAA	GAAAGAGACCATTCCGCCCA
*Aco1*	ATCCAGACTTCCAACATFAG	AACCACATGATTTCTTCAGG
*Adrb3*	TTGTCCTGGTGTGGATCGTG	TTGGAGGCAAAGGAACAGCA
*Atgl*	TTCACCATCCGCTTGTTGGAG	AGATGGTCACCCAATTTCCTC
*Cd137*	GGTCTGTGCTTAAGACCGGG	TCTTAATAGCTGGTCCTCCCTC
*Cebpa*	AGGTGCTGGAGTTGACCAGT	CAGCCTAGAGATCCAGCGAC
*Cidea*	CGGGAATAGCCAGAGTCACC	TGTGCATCGGATGTCGTAGG
*Cited*	AACCTTGGAGTGAAGGATCGC	GTAGGAGAGCCTATTGGAGATGT
*Cox4*	TGACGGCCTTGGACGG	CGATCAGCGTAAGTGGGGA
*Cpt1*	GTGTTGGAGGTGACAGACTT	CACTTTCTCTTTCCACAAGG
*Fasn*	TTGCTGGCACTACAGAATGC	AACAGCCTCAGAGCGACAAT
*Fgf21*	CGTCTGCCTCAGAAGGACTC	TCTACCATGCTCAGGGGGTC
*Hsl*	GCACTGTGACCTGCTTGGT	CTGGCACCCTCACTCCATA
*Lpl*	AGGACCCCTGAAGACACAGCT	TGTACAGGGCGGCCACAAGT
*Nrf1*	GCTAATGGCCTGGTCCAGAT	CTGCGCTGTCCGATATCCTG
*Pgc* *-* *1a*	ATGTGCAGCCAAGACTCTGTA	CGCTACACCACTTCAATCCAC
*Plin1*	GCAAGAAGAGCTGAGCAGAC	AATCTGCCCACGAGAAAGGA
*Ppara*	GAGAGGGCACACGCTAGGAA	GAACACCAATGTTCGGAGCC
*Pparg*	CAAGAATACCAAAGTGCGATCAA	GAGCTGGGTCTTTTCAGAATAATAAG
*Prdm16*	GATGGGAGATGCTGACGGAT	TGATCTGACACATGGCGAGG
*Prkaa1*	GCGCCATGCGCAGACTCA	GTGTCCCCCAGGATGTAGTGG
*Srebf1*	GCTTAGCCTCTACACCAACTGGC	ACAGACTGGTACGGGCCACAAG
*Tbx1*	AGCGAGGCGGAAGGGA	CCTGGTGACTGTGCTGAAGT
*Tfam*	ATGTGGAGCGTGCTAAAAGC	GGATAGCTACCCATGCTGGAA
*Tmem26*	CCATGGAAACCAGTATTGCAGC	ATTGGTGGCTCTGTGGGATG
*Ucp1*	CCTGCCTCTCTCGGAAACAA	GTAGCGGGGTTTGATCCCAT
*G* *apdh*	TTGTTGCCATCAACGACCCC	GCCGTTGAATTTGCCGTGAG

## Data Availability

The data presented in this research are available from the corresponding authors upon request.

## Supplementary Materials

The following supporting information can be downloaded at: https://www.mdpi.com/article/10.3390/ph18121843/s1, Figure S1: Effects of Extracts of *Coix lacryma-jobi* L. (CLE) and *Raphanus sativus* L. (RSE) on cell viability and lipid accumulation in 3T3-L1 adipocytes; Figure S2: Effects of CLE and RSE on the expression of thermogenic and beige fat-specific markers in 3T3-L1 adipocyte; Figure S3: Effects of CLE and RSE on mitochondrial biogenesis in 3T3-L1 adipocytes; Figure S4: Effects of coixol and sinigrin on the expression of fat browning through signaling pathway validation in 3T3-L1 adipocytes.

## Abbreviations

The following abbreviations are used in this manuscript:

2-βME	2-mercaptoethanol
ACC	Acetyl-CoA carboxylase
ACO1	Acyl-CoA carboxylase 1
AMPK	Adenosine monophosphate–activated protein kinase
ATGL	Adipose triglyceride lipase
ATP	Adenosine triphosphate
BAT	Brown adipose tissue
BMI	Body mass index
BSA	Bovine serum albumin
C/EBP	CCAAT/enhancer binding protein
CD137	Tumor necrosis factor receptor superfamily, member 9
CIDEA	Cell death-inducing DNA fragmentation factor alpha-like effector A
CITED	Cbp/p300-interacting transactivator, with Glu/Asp-rich carboxy-terminal domain
CLE	Extracts of *Coix lacryma-jobi* L.
COX4	Cytochrome c oxidase subunit 4
CPT1	Carnitine palmitoyltransferase 1
Ct	Cycle threshold
DAPI	4′,6-Diamidino-2-phenylindole
DEX	Dexamethasone
DMEM	Dulbecco’s modified eagle’s medium/high glucose
DMSO	Dimethyl sulfoxide
DW	Deionized water
FASN	Fatty acid synthase
FBS	Fetal bovine serum
FITC	Fluorescein isothiocyanate
GAPDH	Glyceraldehyde-3-phosphate dehydrogenase
GLP-1	Glucagon-like peptide 1
HSL	Hormone-sensitive lipase
IBMX	3-isobutyl-1-methylxanthine
LPL	Lipoprotein lipase
MTT	3-(4,5-dimethylthiazol-2-yl)-2,5-diphenyltetrazolium
NCS	Newborn bovine calf serum
NRF1	Nuclear respiratory factor 1
PBS	Phosphate-buffered saline
PGC-1α	Peroxisome proliferator-activated receptor gamma co-activator 1α
P-S	Penicillin-Streptomycin solution
PKA	Protein kinase A
PLIN	Perilipin
PPAR	Peroxisome proliferator-activated receptor
PRDM16	PR domain containing 16
qRT-PCR	Quantitative real-time polymerase chain reaction
RSE	Extract of *Raphanus sativus* L.
SEM	Standard error of the mean
TBX1	T-box 1
TFAM	Mitochondrial transcription factor A
TJT	*Taeeumjowi**-**tang*
TMEM26	Transmembrane Protein 26
TNF	Tumor necrosis factor
UCP1	Uncoupling protein 1
USA	United States
WAT	White adipose tissue
WHO	World Health Organization
β3-AR	Beta 3 adrenergic receptor

## References

[1] Marti A., Bjorkman S.H., García-Peña L.M., Weatherford E.T., Jena J., Pereira R.O. (2025). ATF4 Deletion in Brown Adipocytes Attenuates Diet-Induced Insulin Resistance in Male Mice Independently of Weight Gain. Endocrinology.

[2] Gu Y., He W., Li W., Cai J., Wang Z., Li K., Qin G., Gu X., Lin X., Ma L. (2025). Arctiin, a Lignan Compound, Enhances Adipose Tissue Browning and Energy Expenditure by Activating the Adenosine A2A Receptor. Mol. Med..

[3] Guo Y.-Y., Li B.-Y., Peng W.-Q., Guo L., Tang Q.-Q. (2019). Taurine-Mediated Browning of White Adipose Tissue Is Involved in Its Anti-Obesity Effect in Mice. J. Biol. Chem..

[4] Zhang H., Xiong P., Zheng T., Hu Y., Guo P., Shen T., Zhou X. (2025). Combination of Berberine and Evodiamine Alleviates Obesity by Promoting Browning in 3T3-L1 Cells and High-Fat Diet-Induced Mice. Int. J. Mol. Sci..

[5] Pahlavani M., Pham K., Kalupahana N.S., Morovati A., Ramalingam L., Abidi H., Kiridana V., Moustaid-Moussa N. (2025). Thermogenic Adipose Tissues: Promising Therapeutic Targets for Metabolic Diseases. J. Nutr. Biochem..

[6] Cannon B., Nedergaard J. (2004). Brown Adipose Tissue: Function and Physiological Significance. Physiol. Rev..

[7] Becher T., Palanisamy S., Kramer D.J., Eljalby M., Marx S.J., Wibmer A.G., Butler S.D., Jiang C.S., Vaughan R., Schöder H. (2021). Brown Adipose Tissue Is Associated with Cardiometabolic Health. Nat. Med..

[8] Kim S.-J., Seo Y.-H., Lee H.-S., Chang H.-K., Cho J.-H., Kim K.-W., Song M.-Y. (2020). Research Trends of Herbal Medicines for Obesity: Mainly since 2015 to 2019. J. Korean Med. Rehabil..

[9] Hsiao M.H., Ko S.-G., Jun C.-Y., Park J.-H., Choi Y.-K. (2010). Effects of Taeumjowe-tang-gagambang on the Glycometabolism and Lipidmetabolism in the Liver Tissue of Diet-induced Obesity Mice. J. Physiol. Pathol. Korean Med..

[10] Kwak J.-Y., Ahn T.-W. (2020). The Effects of Taeeumjowi-tang Extract Granule on Metabolic Syndrome Risk Factors with Obesity: A Single Group, Prospective, Multi-Center Trial. J. Sasang Const. Immune Med..

[11] Li G.-R., Cao B.-H., Liu W., Ren R.-H., Feng J., Lv D.-J. (2020). Isolation and Identification of Endophytic Fungi in Kernels of *Coix Lachrymal-Jobi* L. Cultivars. Curr. Microbiol..

[12] Li H., Peng L., Yin F., Fang J., Cai L., Zhang C., Xiang Z., Zhao Y., Zhang S., Sheng H. (2024). Research on Coix Seed as a Food and Medicinal Resource, It’s Chemical Components and Their Pharmacological Activities: A Review. J. Ethnopharmacol..

[13] Ha D.T., Nam Trung T., Bich Thu N., Van On T., Hai Nam N., Van Men C., Thi Phuong T., Bae K. (2010). Adlay Seed Extract (*Coix Lachryma-Jobi* L.) Decreased Adipocyte Differentiation and Increased Glucose Uptake in 3T3-L1 Cells. J. Med. Food.

[14] Zhao M., Zhu D., Sun-Waterhouse D., Su G., Lin L., Wang X., Dong Y. (2014). In Vitro and in Vivo Studies on Adlay-Derived Seed Extracts: Phenolic Profiles, Antioxidant Activities, Serum Uric Acid Suppression, and Xanthine Oxidase Inhibitory Effects. J. Agric. Food Chem..

[15] Chen L.-C., Jiang B.-K., Zheng W.-H., Zhang S.-Y., Li J.-J., Fan Z.-Y. (2019). Preparation, Characterization and Anti-Diabetic Activity of Polysaccharides from Adlay Seed. Int. J. Biol. Macromol..

[16] Liu H., Li L., Zou J., Zhou T., Wang B., Sun H., Yu S. (2019). Coix Seed Oil Ameliorates Cancer Cachexia by Counteracting Muscle Loss and Fat Lipolysis. BMC Complement. Altern. Med..

[17] Lin P.-H., Shih C.-K., Yen Y.-T., Chiang W., Hsia S.-M. (2019). Adlay (*Coix Lachryma-Jobi* L. *Var. Ma-Yuen* Stapf.) Hull Extract and Active Compounds Inhibit Proliferation of Primary Human Leiomyoma Cells and Protect against Sexual Hormone-Induced Mice Smooth Muscle Hyperproliferation. Molecules.

[18] Wang Y., Zhao X., Wang W., Kan J., Yu Y. (2013). Analysis and Evaluation of Nutritional Components in Different Tissues of Coix Lacryma-Jobi. Food Sci..

[19] Choi C.K., Kim K.J., Ryu S.N., Lee B.H. (1999). HPLC Quantitative Determination of Coixol Component Coix Lachryma-Jobi Var. Mayuen STAPE. J. Korean Soc. Int. Agric..

[20] Cheung F. (2011). TCM: Made in China. Nature.

[21] Chen H.-H., Chiang W., Chang J.-Y., Chien Y.-L., Lee C.-K., Liu K.-J., Cheng Y.-T., Chen T.-F., Kuo Y.-H., Kuo C.-C. (2011). Antimutagenic Constituents of Adlay (*Coix Lachryma-Jobi* L. Var. *Ma-Yuen* Stapf) with Potential Cancer Chemopreventive Activity. J. Agric. Food Chem..

[22] Hameed A., Hafizur R.M., Khan M.I., Jawed A., Wang H., Zhao M., Matsunaga K., Izumi T., Siddiqui S., Khan F. (2019). Coixol Amplifies Glucose-Stimulated Insulin Secretion via cAMP Mediated Signaling Pathway. Eur. J. Pharmacol..

[23] Cui E., Qian S., Li J., Jiang X., Wang H., Du S., Du L. (2023). Discovery of Coixol Derivatives as Potent Anti-Inflammatory Agents. J. Nat. Prod..

[24] Shen X.-Y., Lu J.-M., Lu Y.-N., Jin G.-N., Ma J.-W., Wang J.-H., Wang Y., Xu X., Piao L.-X. (2023). Coixol Ameliorates Toxoplasma Gondii Infection-Induced Lung Injury by Interfering with T. Gondii HSP70/TLR4/NF-κB Signaling Pathway. Int. Immunopharmacol..

[25] Gao L., Li H., Li B., Shao H., Yu X., Miao Z., Zhang L., Zhu L., Sheng H. (2022). Traditional Uses, Phytochemistry, Transformation of Ingredients and Pharmacology of the Dried Seeds of *Raphanus sativus* L. (Raphani Semen), A Comprehensive Review. J. Ethnopharmacol..

[26] Manivannan A., Kim J.-H., Kim D.-S., Lee E.-S., Lee H.-E. (2019). Deciphering the Nutraceutical Potential of Raphanus Sativus-A Comprehensive Overview. Nutrients.

[27] Kim K.H., Kim C.S., Park Y.J., Moon E., Choi S.U., Lee J.H., Kim S.Y., Lee K.R. (2015). Anti-Inflammatory and Antitumor Phenylpropanoid Sucrosides from the Seeds of Raphanus Sativus. Bioorg. Med. Chem. Lett..

[28] Song M.-Y., Kim E.-K., Moon W.-S., Park J.-W., Kim H.-J., So H.-S., Park R., Kwon K.-B., Park B.-H. (2009). Sulforaphane Protects against Cytokine- and Streptozotocin-Induced Beta-Cell Damage by Suppressing the NF-kappaB Pathway. Toxicol. Appl. Pharmacol..

[29] He C., Li B., Song W., Ding Z., Wang S., Shan Y. (2014). Sulforaphane Attenuates Homocysteine-Induced Endoplasmic Reticulum Stress through Nrf-2-Driven Enzymes in Immortalized Human Hepatocytes. J. Agric. Food Chem..

[30] Saleh H.A., Ramdan E., Elmazar M.M., Azzazy H.M.E., Abdelnaser A. (2021). Comparing the Protective Effects of Resveratrol, Curcumin and Sulforaphane against LPS/IFN-γ-Mediated Inflammation in Doxorubicin-Treated Macrophages. Sci. Rep..

[31] Yi G., Lim S., Chae W.B., Park J.E., Park H.R., Lee E.J., Huh J.H. (2016). Root Glucosinolate Profiles for Screening of Radish (*Raphanus sativus* L.) Genetic Resources. J. Agric. Food Chem..

[32] Mazumder A., Dwivedi A., du Plessis J. (2016). Sinigrin and Its Therapeutic Benefits. Molecules.

[33] Fan Q., Xi P., Tian D., Jia L., Cao Y., Zhan K., Sun T., Zhang Y., Wang Q. (2021). Ginsenoside Rb1 Facilitates Browning by Repressing Wnt/β-Catenin Signaling in 3T3-L1 Adipocytes. Med. Sci. Monit. Int. Med. J. Exp. Clin. Res..

[34] Kong L., Zhang W., Liu S., Zhong Z., Zheng G. (2022). Quercetin, Engelitin and Caffeic Acid of *Smilax china* L. Polyphenols, Stimulate 3T3-L1 Adipocytes to Brown-like Adipocytes Via Β3-AR/AMPK Signaling Pathway. Plant Foods Hum. Nutr. Dordr. Neth..

[35] Lee H.S., Heo C.U., Song Y.-H., Lee K., Choi C.-I. (2023). Naringin Promotes Fat Browning Mediated by UCP1 Activation via the AMPK Signaling Pathway in 3T3-L1 Adipocytes. Arch. Pharm. Res..

[36] Lee H.S., Choi S.M., Lim S.H., Choi C.-I. (2023). Betanin from Beetroot (*Beta vulgaris* L.) Regulates Lipid Metabolism and Promotes Fat Browning in 3T3-L1 Adipocytes. Pharmaceuticals.

[37] Choi S.M., Lee H.S., Lim S.H., Choi G., Choi C.-I. (2024). Hederagenin from Hedera Helix Promotes Fat Browning in 3T3-L1 Adipocytes. Plants.

[38] Zhang P., He Y., Wu S., Li X., Lin X., Gan M., Chen L., Zhao Y., Niu L., Zhang S. (2022). Factors Associated with White Fat Browning: New Regulators of Lipid Metabolism. Int. J. Mol. Sci..

[39] Pilkington A.-C., Paz H.A., Wankhade U.D. (2021). Beige Adipose Tissue Identification and Marker Specificity-Overview. Front. Endocrinol..

[40] Pfanner N., Warscheid B., Wiedemann N. (2019). Mitochondrial Proteins: From Biogenesis to Functional Networks. Nat. Rev. Mol. Cell Biol..

[41] Heinonen S., Jokinen R., Rissanen A., Pietiläinen K.H. (2020). White Adipose Tissue Mitochondrial Metabolism in Health and in Obesity. Obes. Rev..

[42] Cho C.H., Patel S., Rajbhandari P. (2023). Adipose Tissue Lipid Metabolism: Lipolysis. Curr. Opin. Genet. Dev..

[43] Lampidonis A.D., Rogdakis E., Voutsinas G.E., Stravopodis D.J. (2011). The Resurgence of Hormone-Sensitive Lipase (HSL) in Mammalian Lipolysis. Gene.

[44] Nielsen T.S., Jessen N., Jørgensen J.O.L., Møller N., Lund S. (2014). Dissecting Adipose Tissue Lipolysis: Molecular Regulation and Implications for Metabolic Disease. J. Mol. Endocrinol..

[45] Cero C., Lea H.J., Zhu K.Y., Shamsi F., Tseng Y.-H., Cypess A.M. (2021). Β3-Adrenergic Receptors Regulate Human Brown/Beige Adipocyte Lipolysis and Thermogenesis. JCI Insight.

[46] Garcia D., Shaw R.J. (2017). AMPK: Mechanisms of Cellular Energy Sensing and Restoration of Metabolic Balance. Mol. Cell.

[47] Steinberg G.R., Hardie D.G. (2023). New Insights into Activation and Function of the AMPK. Nat. Rev. Mol. Cell Biol..

[48] Jensen M.D., Ryan D.H., Apovian C.M., Ard J.D., Comuzzie A.G., Donato K.A., Hu F.B., Hubbard V.S., Jakicic J.M., Kushner R.F. (2014). 2013 AHA/ACC/TOS Guideline for the Management of Overweight and Obesity in Adults: A Report of the American College of Cardiology/American Heart Association Task Force on Practice Guidelines and The Obesity Society. Circulation.

[49] Chandrasekaran P., Weiskirchen R. (2024). The Role of Obesity in Type 2 Diabetes Mellitus-An Overview. Int. J. Mol. Sci..

[50] Lundgren J.R., Janus C., Jensen S.B.K., Juhl C.R., Olsen L.M., Christensen R.M., Svane M.S., Bandholm T., Bojsen-Møller K.N., Blond M.B. (2021). Healthy Weight Loss Maintenance with Exercise, Liraglutide, or Both Combined. N. Engl. J. Med..

[51] Hinte L.C., Castellano-Castillo D., Ghosh A., Melrose K., Gasser E., Noé F., Massier L., Dong H., Sun W., Hoffmann A. (2024). Adipose Tissue Retains an Epigenetic Memory of Obesity after Weight Loss. Nature.

[52] Boström P., Wu J., Jedrychowski M.P., Korde A., Ye L., Lo J.C., Rasbach K.A., Boström E.A., Choi J.H., Long J.Z. (2012). A PGC1-α-Dependent Myokine That Drives Brown-Fat-like Development of White Fat and Thermogenesis. Nature.

[53] Kim S.H., Plutzky J. (2016). Brown Fat and Browning for the Treatment of Obesity and Related Metabolic Disorders. Diabetes Metab. J..

[54] Machado S.A., Pasquarelli-do-Nascimento G., da Silva D.S., Farias G.R., de Oliveira Santos I., Baptista L.B., Magalhães K.G. (2022). Browning of the White Adipose Tissue Regulation: New Insights into Nutritional and Metabolic Relevance in Health and Diseases. Nutr. Metab..

[55] Cheng C.-F., Ku H.-C., Lin H. (2018). PGC-1α as a Pivotal Factor in Lipid and Metabolic Regulation. Int. J. Mol. Sci..

[56] Seale P., Conroe H.M., Estall J., Kajimura S., Frontini A., Ishibashi J., Cohen P., Cinti S., Spiegelman B.M. (2011). Prdm16 Determines the Thermogenic Program of Subcutaneous White Adipose Tissue in Mice. J. Clin. Investig..

[57] Harms M., Seale P. (2013). Brown and Beige Fat: Development, Function and Therapeutic Potential. Nat. Med..

[58] Janani C., Ranjitha Kumari B.D. (2015). PPAR Gamma Gene—A Review. Diabetes Metab. Syndr..

[59] Rosen E.D., Sarraf P., Troy A.E., Bradwin G., Moore K., Milstone D.S., Spiegelman B.M., Mortensen R.M. (1999). PPAR Gamma Is Required for the Differentiation of Adipose Tissue in Vivo and in Vitro. Mol. Cell.

[60] Sarjeant K., Stephens J.M. (2012). Adipogenesis. Cold Spring Harb. Perspect. Biol..

[61] Wu H., Li X., Shen C. (2020). Peroxisome Proliferator-Activated Receptor γ in White and Brown Adipocyte Regulation and Differentiation. Physiol. Res..

[62] Batchuluun B., Pinkosky S.L., Steinberg G.R. (2022). Lipogenesis Inhibitors: Therapeutic Opportunities and Challenges. Nat. Rev. Drug Discov..

[63] Jeon Y.G., Kim Y.Y., Lee G., Kim J.B. (2023). Physiological and Pathological Roles of Lipogenesis. Nat. Metab..

[64] Sheng R., Yan S.M., Qi L.Z., Zhao Y.L. (2015). Effect of the Ratios of Unsaturated Fatty Acids on the Expressions of Genes Related to Fat and Protein in the Bovine Mammary Epithelial Cells. Vitr. Cell Dev. Biol. Anim..

[65] Carreño D., Hervás G., Toral P.G., Castro-Carrera T., Frutos P. (2016). Fish Oil-Induced Milk Fat Depression and Associated Downregulation of Mammary Lipogenic Genes in Dairy Ewes. J. Dairy Sci..

[66] Uota A., Okuno Y., Fukuhara A., Sasaki S., Kobayashi S., Shimomura I. (2024). ARMC5 Selectively Degrades SCAP-Free SREBF1 and Is Essential for Fatty Acid Desaturation in Adipocytes. J. Biol. Chem..

[67] Hiller B., Angulo J., Olivera M., Nuernberg G., Nuernberg K. (2013). How Selected Tissues of Lactating Holstein Cows Respond to Dietary Polyunsaturated Fatty Acid Supplementation. Lipids.

[68] Han S.-F., Jiao J., Zhang W., Xu J.-Y., Zhang W., Fu C.-L., Qin L.-Q. (2017). Lipolysis and Thermogenesis in Adipose Tissues as New Potential Mechanisms for Metabolic Benefits of Dietary Fiber. Nutrition.

[69] Kohlmayr J.M., Grabner G.F., Nusser A., Höll A., Manojlović V., Halwachs B., Masser S., Jany-Luig E., Engelke H., Zimmermann R. (2024). Mutational Scanning Pinpoints Distinct Binding Sites of Key ATGL Regulators in Lipolysis. Nat. Commun..

[70] Pagnon J., Matzaris M., Stark R., Meex R.C.R., Macaulay S.L., Brown W., O’Brien P.E., Tiganis T., Watt M.J. (2012). Identification and Functional Characterization of Protein Kinase A Phosphorylation Sites in the Major Lipolytic Protein, Adipose Triglyceride Lipase. Endocrinology.

[71] Wang Y., Nguyen H.P., Xue P., Xie Y., Yi D., Lin F., Dinh J., Viscarra J.A., Ibe N.U., Duncan R.E. (2024). ApoL6 Associates with Lipid Droplets and Disrupts Perilipin1-HSL Interaction to Inhibit Lipolysis. Nat. Commun..

[72] Zhao T., Ma A., Huang Z., Liu Z., Sun Z., Zhu L., Chang H. (2024). Pparβ Regulates Lipid Catabolism by Mediating Acox and Cpt-1 Genes in Scophthalmus Maximus under Heat Stress. Fish Physiol. Biochem..

[73] Huang Z., Luo R., Yang L., Chen H., Zhang X., Han J., Wang H., Zhou Z., Wang Z., Shao L. (2022). CPT1A-Mediated Fatty Acid Oxidation Promotes Precursor Osteoclast Fusion in Rheumatoid Arthritis. Front. Immunol..

[74] Lin Y., Wang Y., Li P.-F. (2022). PPARα: An Emerging Target of Metabolic Syndrome, Neurodegenerative and Cardiovascular Diseases. Front. Endocrinol..

[75] Collins S. (2022). β-Adrenergic Receptors and Adipose Tissue Metabolism: Evolution of an Old Story. Annu. Rev. Physiol..

[76] Valentine J.M., Ahmadian M., Keinan O., Abu-Odeh M., Zhao P., Zhou X., Keller M.P., Gao H., Yu R.T., Liddle C. (2022). Β3-Adrenergic Receptor Downregulation Leads to Adipocyte Catecholamine Resistance in Obesity. J. Clin. Investig..

[77] Mottillo E.P., Desjardins E.M., Crane J.D., Smith B.K., Green A.E., Ducommun S., Henriksen T.I., Rebalka I.A., Razi A., Sakamoto K. (2016). Lack of Adipocyte AMPK Exacerbates Insulin Resistance and Hepatic Steatosis through Brown and Beige Adipose Tissue Function. Cell Metab..

[78] Jeon H.-J., Choi D.K., Choi J., Lee S., Lee H., Yu J.H., Min S.-H. (2021). D-Mannitol Induces a Brown Fat-like Phenotype via a Β3-Adrenergic Receptor-Dependent Mechanism. Cells.

[79] Lim S., Park J., Um J.-Y. (2019). Ginsenoside Rb1 Induces Beta 3 Adrenergic Receptor–Dependent Lipolysis and Thermogenesis in 3T3-L1 Adipocytes and *Db*/*Db* Mice. Front. Pharmacol..

[80] Chiang H., Lu H.-F., Chen J.-C., Chen Y.-H., Sun H.-T., Huang H.-C., Tien H.-H., Huang C. (2020). Adlay Seed (*Coix Lacryma-Jobi* L.) Extracts Exhibit a Prophylactic Effect on Diet-Induced Metabolic Dysfunction and Nonalcoholic Fatty Liver Disease in Mice. Evid. Based Complement. Altern. Med..

[81] Goyeneche R., Rodrigues C.R., Quispe-Fuentes I., Pellegrini M.C., Cumino A., Di Scala K. (2025). Radish Leaves Extracts as Functional Ingredients: Evaluation of Bioactive Compounds and Health-Promoting Capacities. Waste Biomass Valor..

[82] van Meerloo J., Kaspers G.J.L., Cloos J., Cree I.A. (2011). Cell Sensitivity Assays: The MTT Assay. Cancer Cell Culture: Methods and Protocols.

[83] Jensen E.C. (2013). Quantitative Analysis of Histological Staining and Fluorescence Using ImageJ. Anat. Rec..

