# Supplementary material for: Coixol and Sinigrin from Coix lacryma-jobi L. and Raphanus sativus L. Promote Fat Browning in 3T3-L1 Adipocytes
Source: Pharmaceuticals (Basel). 2025 Dec 2;18(12):1843. doi: 10.3390/ph18121843 (PMC12736028; doi:10.3390/ph18121843)
Supplement: Supplementary file 1 [file pharmaceuticals-18-01843-s001.zip › pharmaceuticals-3947161-supplementary.pdf]

# Supplementary Materials: Coixol and Sinigrin from *Coix lacryma-jobi* L. and *Raphanus sativus* L. Promote Fat Browning in 3T3-L1 Adipocytes

**Seung Min Choi, Sung Ho Lim, Ho Seon Lee, Gayoung Choi, Myeong Ji Kim, Hyunwoo Kim and Chang-Ik Choi**

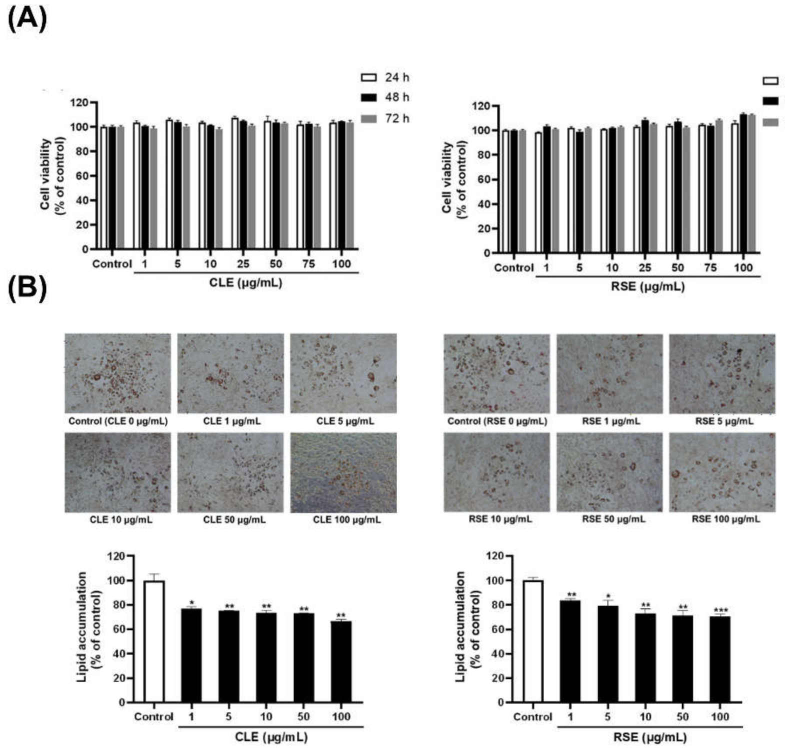


**Figure S1.** Effects of Extracts of *Coix lacryma-jobi* (CLE) and *Raphanus sativus* (RSE) on cell viability and lipid accumulation in 3T3-L1 adipocytes. (A) Cell viability was assessed after 24 h, 48 h, and 72 h of treatment using the MTT assay. Cells were seeded in 96-well plates and incubated for 24 h prior to treatment. Results are expressed as a percentage of the control (0.5% DMSO) (n = 6). Representative cell images were obtained at 100× magnification. (B) Lipid accumulation was evaluated after 7 days of differentiation. Cells were seeded in 24-well plates, with lipid droplets stained with Oil Red O, extracted using isopropanol, and quantified at 520 nm using a microplate reader. Data are expressed as a percentage of the control (0.5% DMSO) and reported as mean ± standard error of the mean (SEM) from triplicates. *$p < 0.05$, **$p < 0.01$, and ***$p < 0.001$ vs. control.

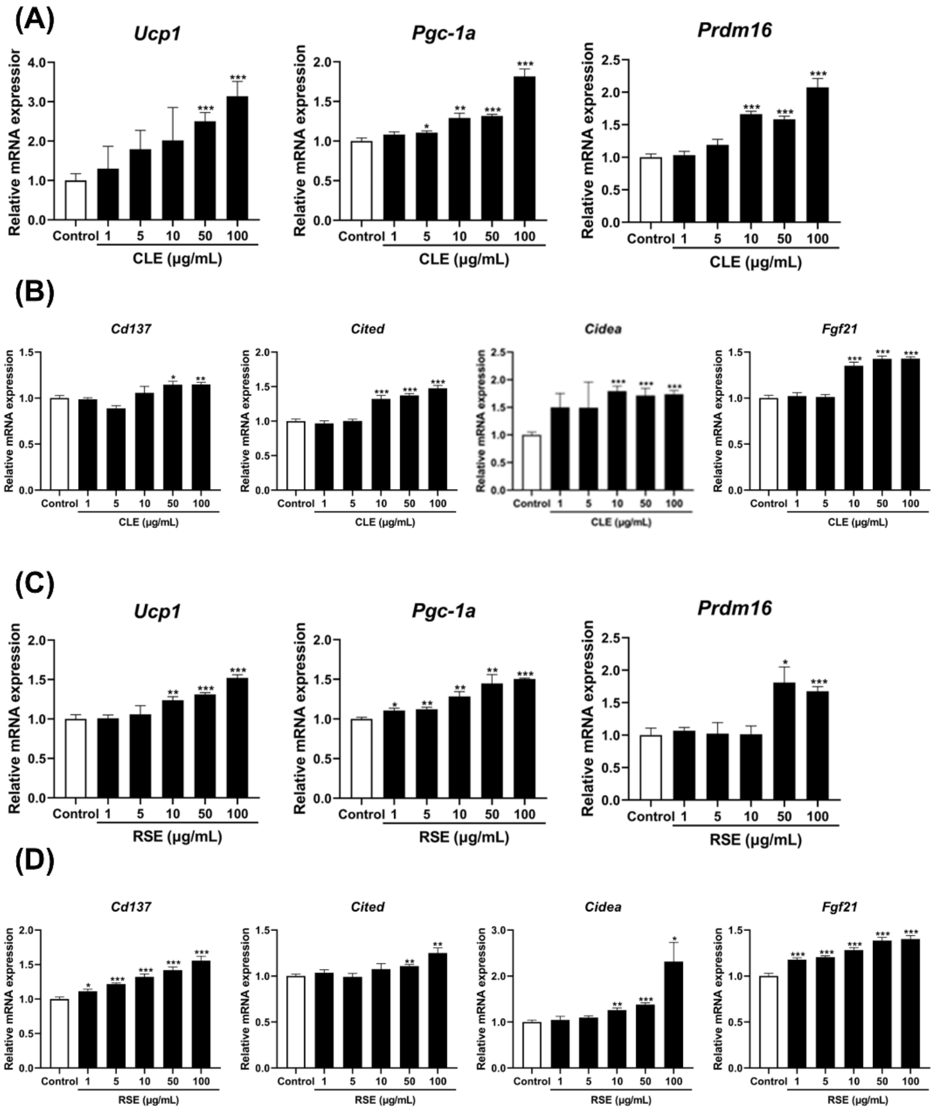


**Figure S2.** Effects of CLE (A, B) and RSE (C, D) on the expression of thermogenic and beige fat-specific markers in 3T3-L1 adipocytes. Target gene mRNA levels were normalized to *Gapdh* using the 2–ΔΔCt method (n = 6). Gapdh was used as the housekeeping gene. Results are presented as mean ± standard error of the mean (SEM). *$p < 0.05$, **$p < 0.01$, and ***$p < 0.001$ vs. control.

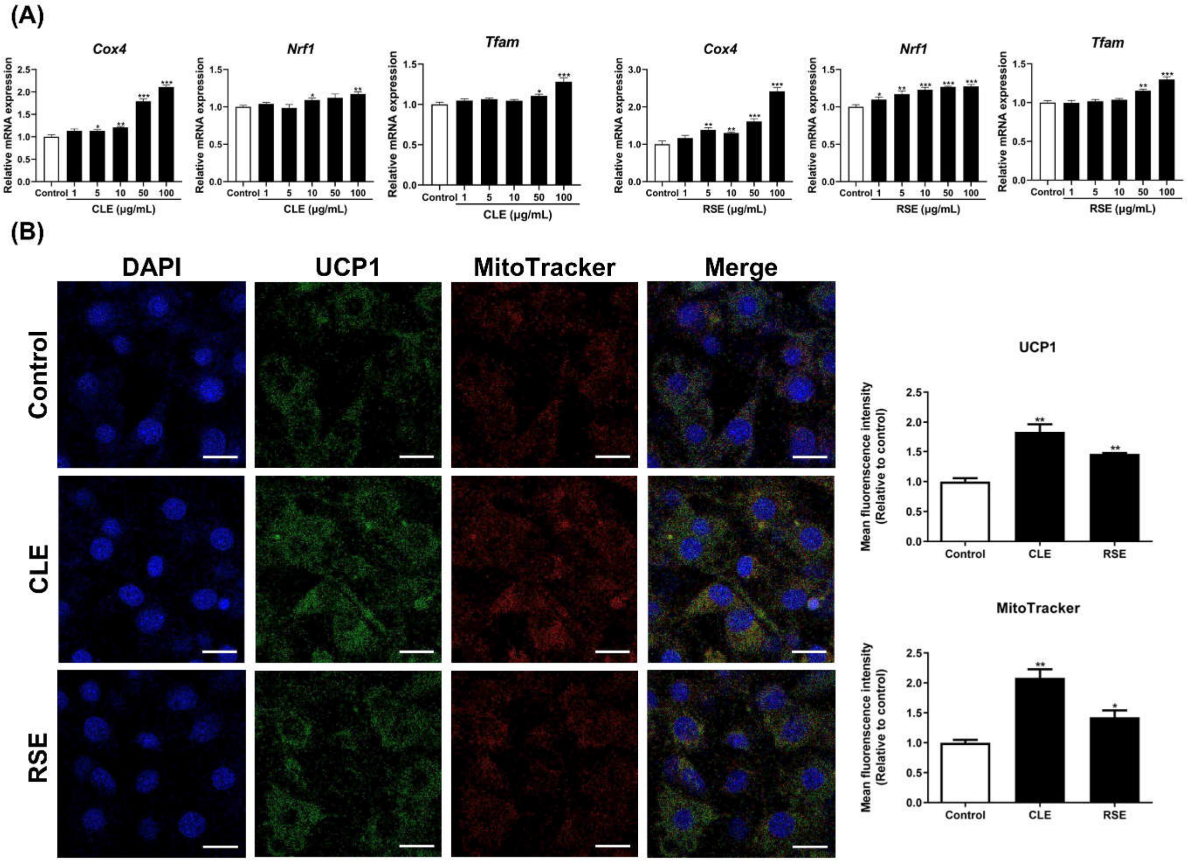


**Figure S3.** Effects of CLE and RSE on mitochondrial biogenesis in 3T3-L1 adipocytes. (A) mRNA expression of mitochondrial biogenesis markers was evaluated using qRT-PCR. *Gapdh* was used as the housekeeping gene, and target gene expression was normalized using the 2−ΔΔCt method. Results are presented as mean ± standard error of the mean (SEM) (n = 6). *$p$ < 0.05, **$p$ < 0.01, and ***$p$ < 0.001 vs. control. (B) Effects of CLE and RSE on intracellular mitochondrial biogenesis, with UCP1 activation evaluated using immunofluorescence staining (n = 3). UCP1 protein localization was visualized using FITC-conjugated antibody (UCP1-FITC), DAPI (nuclei), and MitoTracker Red (mitochondria). Images were obtained at 60× magnification (scale bar = 10 μm).

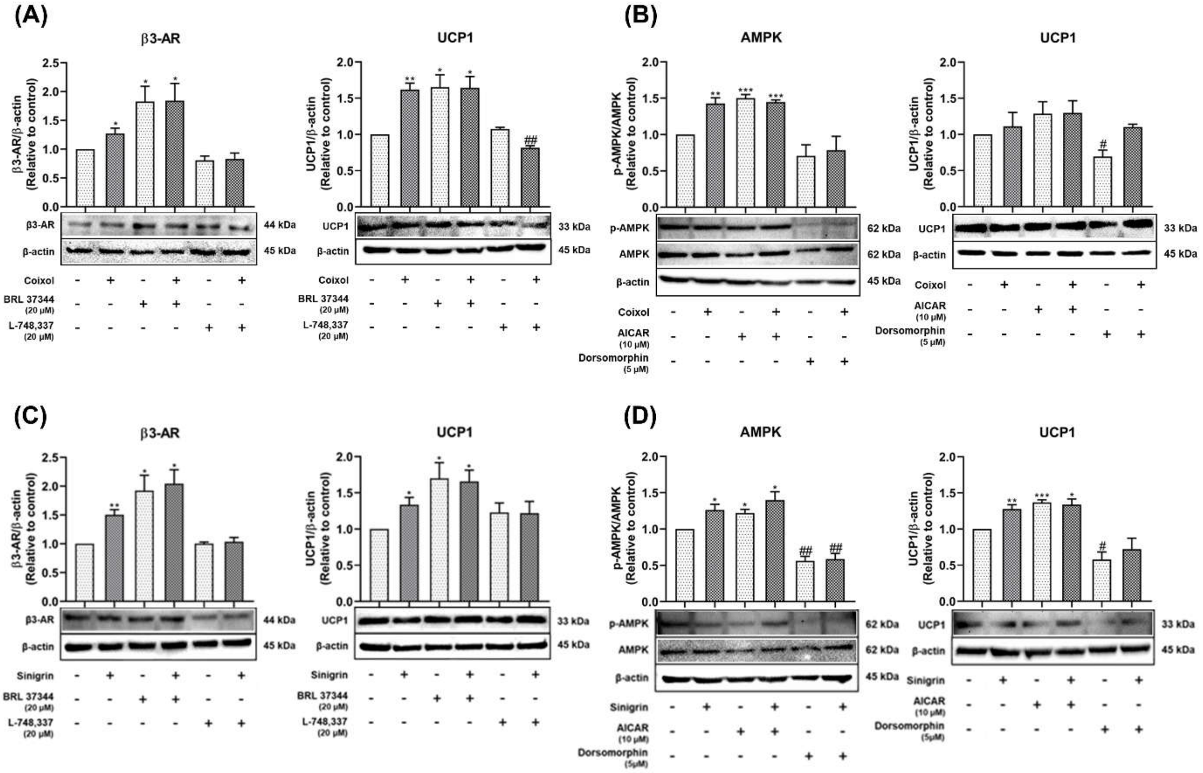


**Figure S4.** Effects of coixol and sinigrin on the expression of fat browning through signaling pathway validation in 3T3-L1 adipocytes. β-actin was used as a loading control for protein analysis. (A, C) Cells were co-treated with coixol or sinigrin together with a β3-AR agonist (BRL 37344, 20 μM) or β3-AR antagonist (L-748,337, 20 μM). (B, D) Cells were co-treated with coixol or sinigrin along with an AMPK activator (AICAR, 10 μM) or AMPK inhibitor (dorsomorphin, 5 μM). Results are presented as mean ± standard error of the mean (SEM) (n = 3). *$p < 0.05$, **$p < 0.01$, and ***$p < 0.001$ compared with the control (increase); #$p < 0.05$ and ##$p < 0.01$ compared with the control (decrease).
